# Patient participation in cancer network governance: a six-year case study

**DOI:** 10.1186/s12913-021-06834-1

**Published:** 2021-09-07

**Authors:** Dominique Tremblay, Nassera Touati, Susan Usher, Karine Bilodeau, Marie-Pascale Pomey, Lise Lévesque

**Affiliations:** 1 Centre de recherche Charles-Le Moyne - Saguenay–Lac-Saint-Jean sur les innovations en santé, Campus de Longueuil - Université de Sherbrooke, 150 Place Charles LeMoyne - Bureau 200, Québec J4K 0A Longueuil, Canada; 2 École Nationale d’Administration Publique, 4750 ave Henri-Julien, 5e étage, Québec H2T 3E5 Montréal, Canada; 3 Faculty of Nursing, Université de Montréal, Pavillon Marguerite-d’Youville, local 7101, 2375 chemin de la Côte-Ste-Catherine, Québec H3T 1A8 Montréal, Canada; 4 School of Public Health, Université de Montréal, 7101 ave du Parc, 3e étage, bureau 3014-8, Québec H3N 1X9 Montréal, Canada

**Keywords:** Patient participation, Cancer, Networks, Governance functions, Collaborative governance, Multiple case study design, Qualitative data

## Abstract

**Background:**

Patient participation in decision-making has become a hallmark of responsive healthcare systems. Cancer networks in many countries have committed to involving people living with and beyond cancer (PLC) at multiple levels. However, PLC participation in network governance remains highly variable for reasons that are poorly understood. This study aims to share lessons learned regarding mechanisms that enable PLC participation in cancer network governance.

**Methods:**

This multiple case study, using a qualitative approach in a natural setting, was conducted over six years in three local cancer networks within the larger national cancer network in Quebec (Canada), where PLC participation is prescribed by the Cancer Directorate. Data were collected from multiple sources, including individual and focus group interviews (*n* = 89) with policymakers, managers, clinicians and PLC involved in national and local cancer governance committees. These data were triangulated and iteratively analysed according to a framework based on functions of collaborative governance in the network context.

**Results:**

We identify three main mechanisms that enable PLC participation in cancer network governance: (1) consistent emphasis on patient-centred care as a network objective; (2) flexibility, time and support to translate mandated PLC representation into meaningful participation; and (3) recognition of the distinct knowledge of PLC in decision-making. The shared vision of person-centred care facilitates PLC participation. The quality of participation improves through changes in how committee meetings are conducted, and through the establishment of a national committee where PLC can pool their experience, develop skills and establish a common voice on priority issues. PLC knowledge is especially valued around particular challenges such as designing integrated care trajectories and overcoming barriers to accessing care. These three mechanisms interact to enable PLC participation in governance and are activated to varying extents in each local network.

**Conclusions:**

This study reveals that mandating PLC representation on governance structures is a powerful context element enabling participation, but that it also delineates which governance functions are open to influence from PLC participation. While the activation of mechanisms is context dependent, the insights from this study in Quebec are transferable to cancer networks in other jurisdictions seeking to embed PLC participation in decision-making.

**Supplementary Information:**

The online version contains supplementary material available at 10.1186/s12913-021-06834-1.

## Background

Efforts to enable participation of people living with and beyond cancer (PLC) are emphasized in many countries to guide the delivery and improvement of cancer care [1–4]. Many terms exist to describe this relationship between patients and health systems components (e.g. patient partnership, patient empowerment, patient centeredness) [5]. A practical definition of PLC participation in governance refers to a process that is attentive to the experiential knowledge and expertise of PLC alongside that of providers and managers. It involves a form of collaborative governance, where authority and responsibility are distributed within the network, rather than traditional centralized notions of governance [6], and requires that the PLC voice be present from macro level strategic planning to micro level service delivery in the cancer network.

The literature suggests that patient participation can provide a unique 360^o^ view of care processes and encourage collaboration in decision-making [7, 8], notably by breaking down hierarchical barriers among providers [9, 10] as well as between providers and PLC [11]. Patient participation manifests concretely in a variety of activities that enable mutual exchange of experience and knowledge between patients (and/or families or patient associations), healthcare providers and policy-makers to shape health and social care services [12]. There is some evidence that patient participation can enhance healthcare governance [9], and recent interest in patient influence on governance in the network context [13], but little evidence about how to enable participation in the particular area of cancer networks. While PLC participation and network governance may appear as connected concepts, how to translate them into practice needs more considerations [5]. This multiple case study aims to share lessons learned regarding underlying mechanisms that enable PLC participation in cancer network governance.

### PLC participation in the Quebec cancer network

The Quebec Cancer Network, led by a Cancer Directorate within the Ministry of Health and Social Services (MSSS) represents a typical case to better understand PLC participation. Cancer is a major and highly visible part of the province’s publicly funded healthcare system. The network-based structure is a key component of the National Cancer Plan, launched in 1998 to coordinate the organisation and delivery of integrated care [14]. In 2017, the Cancer Directorate published a framework for partnering with PLC, specifying that local committees were expected to recruit PLC, assure training and support, equip them with documentation needed to participate in committee deliberations, and reserve a dedicated item on the meeting agenda for PLC contributions [15]. However, PLC participation has been variable between the different local committees, and gaps persist between the vision set out by the Cancer Directorate and practice across the network.

**Fig. 1:**
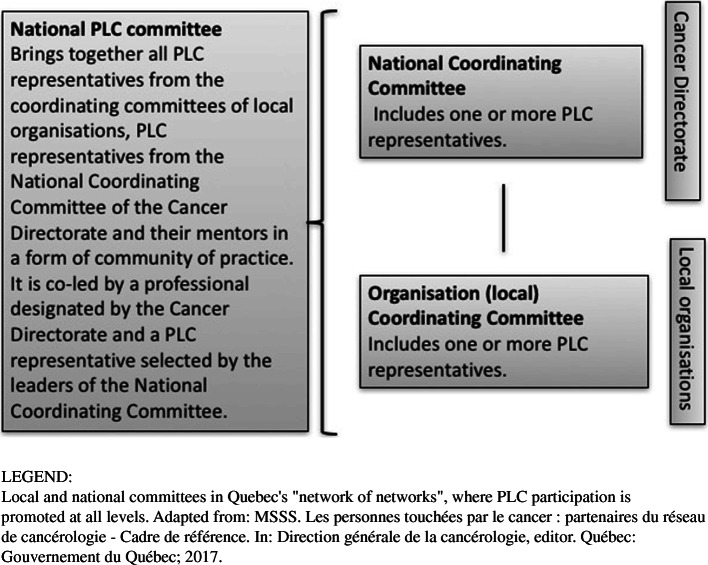
PLC representation in the Quebec Cancer Network

PLC participation in cancer network governance is prescribed by policymakers at national level, but operationalized at organisational and clinical level. A “network of networks” (national and local levels) is the overall organisational model, in which PLC participation is promoted at all levels (Fig. 1) [16]. At the local level, Organisation Coordinating Committees co-chaired by medical and clinical managers are meant to include PLC along with professional, medical, public health and non-profit service providers involved in the cancer trajectory. At national level, two PLC participate alongside clinicians and managers from each local organisation in a National Coordinating Committee. These two committees date back to 2013, while a third, the national PLC Committee, was established in 2017 to bring together, in a form of community of practice, PLC representatives from local and national coordinating committees to expand patient (and family) participation in cancer program governance processes. In practice, network-based working is suited to addressing complex problems that cannot be solved or even formulated in a definitive way. While such problems increase pressure for coordination, network dynamics intensify uncertainty and pose challenges to classic top-down governance [17].

### Conceptual framework

This case study is based on a conceptual framework constructed around governance and how it is expected to work in a network with formalized structures for PLC participation. A classic definition of governance refers to “the conduct of collective action from a position of authority” ([18], p. 31). Denis et al. [19] define five governance functions, which have been adapted to cancer networks [20]: (1) formulating a mission and vision of durable high-quality care in the network; (2) allocating resources to achieve network goals; (3) managing relationships to foster connections between individuals and organisations in the network; (4) managing knowledge to support cancer network integration, and (5) monitoring and control. This definition helps to describe the governance activities in which PLC are involved. Network-based functioning tends to democratise governance, with collaborative governance regimes [21] that support interactions among service users, government decision-makers and health service providers [22]. The coordination of complex relationships between multiple individuals and organisations in cancer networks is meant to enable movement from bureaucratic, siloed and rigid structures to cross-boundary learning, shared decision-making, collective capacity for action, and ultimately, care and services that are responsive to the needs of PLC [23]. PLC participation in cancer network governance can be seen as part of a major shift from coordination undertaken from a unique position of authority, to collaborative regimes [21] that foster motivation, engagement and joint activity among stakeholders with different perspectives [22].

### Knowledge to practice gap

Research in European cancer systems finds wide variation between countries in opportunities for PLC participation [24]. A study of cancer networks in England shows that the experiential knowledge of PLC, while increasingly present, remains peripheral to the elite scientific apparatus in decision-making [25]. Efforts to engage patients in defining what they need from care and undertaking improvement are still “in their infancy” [26]. This study seeks to understand underlying mechanisms that enable PLC participation in - and this implies influence on - governance. Mechanisms refer to “underlying entities, processes, or structures which operate in particular contexts to generate outcomes of interest” ([27], p. 368): in this case PLC participation in governance of the Quebec cancer network.

## Methods

We conducted a qualitative multiple case study to elucidate mechanisms that enable PLC participation in the network governance structures of the Quebec Cancer Network.

### Study design

Multiple case study design was chosen as a way to gain in-depth, longitudinal understanding of PLC participation as it unfolds over time within the natural context of the cancer network. The study was conducted over six years (2014 to 2020), to describe interrelated, contextually bounded activities and to discern patterns [28] that point to underlying mechanisms enabling PLC participation in governance functions.

### Case selection

The study of PLC participation in governing a “network of networks” involves a case-within-case approach [29], respectively referring to the national cancer network and the local cancer networks. It provides the opportunity to identify differences and similarities across local cancer networks, and shed light on variations between the PLC participation prescribed by the Cancer Plan and real-world practices. While purposive selection of the three local cases rests on a theoretical rationale, it also reflects the multiple realities of local cancer networks seen across the Quebec cancer network. Selection criteria considered population, territory, urban/rural context, academic or community mission, time since local cancer network implementation, and range of cancer services offered locally [20]. Case selection sought to enlarge the perspective of the phenomenon in context (the cases within the case) and increase the credibility and reliability of findings [28].

### Study procedure

Collaborators at each of the three study sites supported recruitment for interviews and focus groups, informing local actors of the study objectives, and providing the research team with contact information of interested actors. Inclusion criteria were that participants be involved in governance and improvement committees. Participants from the national level were actors in senior positions in government and national agencies identified from public documents and networking. Two people at national level declined the invitation to be interviewed.

### Data collection

Qualitative data were collected from multiple sources (documents, meeting observation, individual interviews, focus groups) and were triangulated [28] to explore PLC participation from multiple perspectives across local and national levels of the network. Documents (n total = 569) from national and local cancer programs included organisational charts, minutes of meetings and notes from non-participant observation of meetings of local and national coordinating committees and the national PLC committee (*n* = 112 between 2015 and end 2019).

Interview and focus group participants were purposefully selected [30] knowledgeable informants [31] with experience of network governance dynamics in local and national governance committees. Participants represented a diversity of perspectives – policymakers, managers, clinician-managers, clinicians and PLC. Participants from different local networks were included to increase the potential for identifying pattern consistencies and divergences that would help to understand what enabled PLC participation in governance functions. Individual interviews were conducted in private offices reserved at the cancer clinic or in the offices of interview participants. Interview and focus group guides (Additional file 1) were built around governance functions [19] and collaborative governance regimes in cancer networks [21] according to the study framework. Individual interviews were conducted with policymaker, manager, clinical manager and clinician participants as well as with PLC participants on the national level committee, while focus groups were held with PLC participants on local committees. Individual interview grids were adapted to the type of participant to explore the dynamics experienced within committees; focus groups (5–6 PLC in each), sought to stimulate discussion and benefit from a range of PLC perspectives regarding participation at local level. Interviews lasted on average 60 min and focus groups 90 min. The individual interviews were conducted across the study period by two researchers (DT and NT) with experience in and knowledge of the cancer network. The focus groups were led by DT with one research team member as note-taker to supplement recording [32] and were held in meeting rooms within the cancer clinics of participating local networks between January and March 2018.

Interview and focus groups recordings were transcribed and data from all sources were integrated into a database managed with QDA Miner™ software [33] and stored with appropriate security measures (de-identified data, password protection, restricted access, firewalls).

### Data analysis

A pragmatic and flexible approach was adopted for data condensation [34] in order to answer the two following questions: How does mandated PLC participation in governance take shape in practice in Quebec’s cancer network? What are the mechanisms that enable PLC participation in cancer network governance? Three coding cycles were performed to move from raw data collected in the field to the identification of mechanisms that allow PLC participation to take shape. An iterative process was used to code segments of raw data illustrative of PLC participation dynamics (first cycle), group site-based (intra-case) and general (inter-case) context elements [28], and consider the interplay between national and local levels. Emerging themes were then categorised in light of the conceptual framework using display matrices (second cycle), and recurring themes were aggregated around main underlying mechanisms that enabled PLC participation in cancer network governance (third cycle) [28].

In the first cycle, two authors (LL and SU) independently coded data from each site. Initial codes were based on interview questions, and the coding tree was expanded to include emergent insights [34]. Coding sought to capture actors’ perspectives on how PLC participation takes shape in governance functions (mission, resource allocation, managing relationships and knowledge, monitoring and control) and collaborative governance dynamics (engagement, mutual understanding, joint activity) in local and national coordinating committees. Codes were then categorized (second cycle) using display matrices, with data illustrative of these thematic categories extracted from document excerpts and interview transcripts.

The third cycle involved looking for recursive patterns and aggregating thematic categories [2] into mechanisms that enabled (and sometimes limited) PLC participation. Each cycle involved much discussion among researchers (DT, NT, SU, LL) to resolve differences in interpretation [35]. The research team brought extensive knowledge of the evolution and challenges of cancer network governance, and insight into both local and national perspectives, to arrive at consensus in the analysis [36].

## Results

A total of 89 participants involved in governance committees participated in the study. Of the 20 participants at national level, seven held senior positions in government, 10 worked in national agencies, and three were PLC on the National Coordinating Committee. Among the 69 participants in committees in the three local cancer networks, 16 were PLC and 53 were managers, clinical managers and clinicians. The mean age of provider participants (national and local) was 52.3 years; 54 % were men and 46 % were women, and a majority had been working with PLC for between five and 10 years. Among PLC participants, 79 % were women and 21 % were men and the mean age was 62.3 years; all but one (spouse of a cancer survivor) were cancer survivors, and almost all had been diagnosed between five and 10 years before joining the study; three were under active treatment for a recurrence or new cancer. The selection criterion for PLC was involvement in local committees. All potential PLC participants who were referred to the team by local collaborators accepted to participate in the focus groups.

In line with the specific study objective, findings across local and national levels highlight three main mechanisms as especially important to enabling PLC participation in network governance functions: (1) consistent emphasis on patient-centred care as a network objective, (2) flexibility, time and support to translate the mandate for PLC representation into meaningful participation, and (3) recognition of the distinct knowledge of PLC in decision-making. This section explores each of these in turn, with Table 1 presenting the process of moving from data to recurring themes that point to these underlying mechanisms. Conditions enabling their activation relate primarily to network structure. The Cancer Directorate as lead organisation at the policy level encourages a distributed and collective approach, including PLC, to some, but not all, governance functions. Resource allocation and monitoring functions are held centrally, beyond reach of the spaces established for PLC participation. Certain paradoxes emerge between mechanisms for PLC participation and network structure.

**Table 1:** Systematic data condensation^**a**^ process

Real-world natural context → Moving from raw data to more theoretical description → Abstraction		
**CODE** ^b^	**CATEGORY** ^c^	**THEME** ^d^
Initial Cancer Program development includes people living with and beyond cancer (PLC) and caregivers. The Cancer Program emphasizes principles of patient-centred care (PCC) and services (document-MSSS 1998 Cancer Program)	Cancer Program promotes PCC	Mechanism 1. Consistent emphasis on patient-centred care as a network objective
“We used to speak of continuous improvement in administrative terms to clinicians and that did not get through to them, not at all. Then **we changed our discourse**, saying: “What we all want is to improve services for the patient, to respond to patient needs, at the right time, for the right person.” **When we talk that way, we reach everyone**” (manager, local level).	PCC is integrated into the values of network actors	
“When **a PLC is at the table**, committee decisions are made in terms of access to quality care. If the PLC is not there, debate can get caught up in concerns such as the status of the establishment or making things easier for providers” (clinical manager, national level).[Committee members were discussing purchase of a $10 million linear accelerator, when…] “the PLC said ‘after I was discharged home, I would have liked to be able to call the nurse.‘ That **brought everyone back down to earth”** (manager, local level).	PLC participation enables providers to understand what PCC means in particular contexts	
Patients on local committees clearly saw how their experience of care enabled improvements to target patient needs and adjust the actions of various providers (PLC on local coordinating committees, focus groups).“**The focus on patient experience** forced us as a community to work on concerted action” (clinical manager, local level).	Providers are motivated to solve problems raised by PLC	
The Cancer Directorate framework for PLC participation emphasizes co-creation and obligation for all local committees to include PLC. “Meeting the Cancer Directorate’s objective of patient- and family-centred care requires real partnership with PLC, notably through **PLC participation on coordinating committees**.“ (Document - MSSS 2017)	The Cancer Directorate mandates inclusion of PLC on national and local committees	Mechanism 2. Flexibility time and support to shape the mandate for PLC representation into meaningful participation
“No one can define the role. You attend and observe and **eventually find your place**. My experience has tipped the balance more than once” (PLC on local committee, from national PLC committee meeting)	Integrating PLC into committee deliberations requires learning on all sides	
“So (they would ask), ‘what do you think?‘ They gave me feedback on what I said. They informed me about follow-up. **My name is on the agenda**. The vocabulary was difficult at first but now it’s fine” (PLC, local level, national PLC committee meeting).	Strategies are developed to enable PLC and other committee members to work together on governance issues	
A national PLC Committee is created and supported by the Ministry to bring together PLC from local committees, **strengthen their voice and develop common tools**. PLC wanted a “kind of super-PLC committee (so that we) can speak to the top levels, influence the top levels (PLC representation, national level)“What they (the PLC members on our committee) were saying **was listened to** because it resonated with what we were hearing from the national PLC committee” (clinical manager, local level).	A protected space is established for collective reflection and confidence building among PLC, where they can develop supports and identify common priorities	
“She (the PLC) makes us aware of issues, but as **our local committee does not make resource allocation decisions**, she is not involved in (that) decision-making (clinical manager, local level)“**We don’t have access to the network operational statistics** and we need to if we want to fully contribute to analysis of what is done and suggest solutions that make sense” (PLC, national level).	Limited PLC influence on resource allocation and monitoring that are not the purview of local coordinating committees	
“Patients are much better informed today and the system needs to **reinforce their strengths and support their weaknesses”** (clinical manager, local level).	PLC and providers construct projects to equip PLC to understand and use services more effectively	Mechanism 3. Recognition of the distinct knowledge of PLC in decision-making
The Cancer Program mandated development of **care trajectories** by tumour site, building on a lung cancer trajectory pilot project in 2014 (Cancer Plan, Cancer Directorate, 2017).“Mapping patient experience along a trajectory **revealed gaps between the reality perceived by providers and that lived by patients”**. These contradictions encouraged providers to re-examine processes and priorities for improvement, and local context assumed greater importance. It also became evident that PLC participation needed to extend beyond hospitals to cover other steps in the care trajectory (clinical manager, local level).	Projects where PLC contribute unique knowledge heighten their role in governance	

LEGEND:

^a^Adapted from Miles, Huberman & Saldana, 2018. *Qualitative data analysis: A methods sourcebook*: Sage Publications, Thousand Oaks

^b^First coding cycle: illustrative document extracts and participant quotes on PLC participation in governance functions and collaborative governance dynamics → CODE

^c^Second coding cycle: recursive patterns around PLC participation in network governance revealed through matrix (code by case) → CATEGORY

^d^Third coding cycle: Essential mechanisms that enable PLC participation in cancer network governance in the Quebec cancer network → THEME

### Mechanism 1: consistent emphasis on patient-centred care as a network objective

The national cancer plan [16] clearly expresses patient-centred care (PCC) as a core value, and the spread of this value is evident in the policies and actions that structure the network at various levels. PCC appears, among study participants at national and local level, as a convergence point coherent with both the clinician’s duty to respond to the individual needs of PLC and the organisation’s mandate to ensure responsiveness to whole person needs. The emphasis on PCC facilitates acceptance of PLC participation in governance to guide decision-making within the network. Some informants note a shift in organisational culture and discourse that promotes collaboration and helps defuse conflict around where services should be located and the roles of different providers.

On committees, PLC embody the idea of patient-centred care as well as clarifying what it means in particular contexts, thereby participating in the governance function of shaping network vision. PLC input emphasizes a broader view of cancer care that includes the survivorship course, primary care and community support services, moving from a perspective of “*conquering cancer*” to “*living with and beyond cancer”*. It widens the spectrum of relevant services and *“reveal(s) gaps between patient and provider perceptions of what constitutes good care”* (clinical manager, local level). When providers see that a solution to a dysfunction in network coordination or patient access is within their control, they are often motivated to solve the problem promptly. For example, to improve PLC access to community support services, one local committee integrated a referral process to 20 community services into the organisation’s electronic record system.

Informants recognise that providers and patients struggle to address multiple wide-ranging priorities around what is needed to optimize the cancer care continuum. A PLC priority such as being able to contact the oncology nurse following discharge therefore lands on the committee table alongside a clinician priority to invest in a $10 million linear accelerator for radiation therapy. Many clinicians and managers express that PLC need to be present on committees to raise issues that would otherwise go unnoticed: *“if something is not named, it’s as though it doesn’t exist”* on the agenda of decision-makers (clinical manager, local level). The experiential knowledge of PLC with utilization of health services can thus be mobilized rapidly. PLC participation *“serves as an important lever to put actions in place”* (clinical manager, local level) and “*forces providers to rethink their positions”* (clinician, local level) when these diverge from PLC perspectives.

Consistent emphasis on patient-centred care as a network objective therefore appears as a mechanism enabling PLC participation in governance: it becomes a common goal that network actors, including PLC, are then interested in working towards collaboratively.

### Mechanism 2: flexibility, time and support to translate mandated PLC representation into meaningful participation

The Cancer Directorate framework mandated that PLC be invited onto local committees, but did not define particular modalities or the roles and responsibilities PLC would assume. There was no preconceived way of working with PLC in network governance structures: “*we’re learning how to swim once we’re in the water*” (policymaker, national level). The Cancer Directorate and national committee looked to PLC for advice on how to shape these opportunities for PLC participation.

Integrating PLC input into deliberations required learning on all sides. Some PLC had difficulty raising problems, felt their input was discounted, notably by physicians, who *“felt they knew what was best”* (PLC, national level) or that there was little place for PLC contributions on the issues discussed. There were also difficulties recruiting PLC on some local committees. Certain PLC informants highlighted that committees were sometimes ill prepared to integrate them, and expressed feeling very alone and isolated at initial meetings (observation notes, national PLC committee meeting). Others considered the meetings were structured to make room for their specific contributions. It took time and effort on all sides to develop strategies to work together on governance issues and establish the legitimacy of PLC participation.

PLC participation in local governance committees was enhanced by the establishment, part way through the study period, of a national PLC committee. It functioned as a community of practice, with representatives from each local network committee and the national committee. The idea came from a PLC on the National Coordinating Committee, who saw the need to provide a protected space where PLC could collectively reflect on issues arising within their respective committees. The Cancer Directorate confirmed the community of practice’s official mandate as to promote communication and sharing of knowledge and experience in order to contribute to improving cancer care and services. It was also made responsible for developing supports for PLC on governance committees [15]. PLC reported that they were better able to contribute to local committees as a result of their participation in this community of practice, a perception that was shared by clinician managers on local committees.

Overall, the national PLC committee increased the confidence and skills of PLC participants, heightened their legitimacy at local level, and provided a national hub to work on common PLC priorities. Physicians’ skill in “breaking bad news” was identified as a first shared priority, and a training program for physicians was developed for province-wide implementation (meeting observation notes and documents, national PLC committee). Providers on local committees also regarded PLC reports of discussions they had in the community of practice as *“a precious source of information about how care is experienced in the regions*” (clinical manager, local level) that complemented professional perspectives. One PLC described the various network committees as a “*mountain chain*”, providing a route for issues arising locally to make their way up to national level: “*That makes it more difficult to pretend that everything is working well when in fact it isn’t*” (PLC, local level).

While national and local coordinating committees and the national PLC committee provide opportunities for participation, they live within a network where decision-making is perceived as highly centralized around the Cancer Directorate. A number of informants expressed a lack of mutual listening between committees and the Directorate, which *“solicits input from the various committees, but does not act on their recommendations*” (clinical manager, local level).

The Cancer Directorate’s centrality enables the embedding of PLC participation on governance committees, but also delimits the governance functions they can influence. Governance functions of resource distribution and monitoring remain at central level. PLC do not yet have access to the dashboards the Ministry is developing, which is regarded as an impediment to participating in governance (observation notes, national PLC committee).

In summary, flexibility, time and support are essential to translating the Directorate’s mandate into meaningful PLC participation at multiple levels and appear as a second mechanism enabling PLC participation in network governance. The establishment of a national PLC committee enables sharing of best practices across the network: PLC gain confidence and collaborate on common priorities, which increases their legitimacy as participants in governance in the eyes of clinician/manager members of local committees.

### Mechanism 3: recognition of the distinct knowledge of PLC in decision-making

Informants at local level recognize that a collaborative approach is needed for PLC to be comfortable sharing their knowledge within committee discussions and for clinicians and managers to accept PLC input as valuable and constructive. PLC participation in decision-making based on experiential knowledge and insight is seen to help defuse controversies among providers on certain issues, enabling a better management of relationships between network actors: providers are less willing and able to defend their interests (such as maintaining a specialized service at a local hospital) when these are misaligned with PLC perspectives (that they are willing to travel to assure quality services). Oftentimes, such discussions lead to new understanding of what patient-centred quality care entails.

A number of initiatives where PLC were most active revolved around improving people’s ability to use services effectively. On local committees, PLC raised concrete issues they faced in meeting their needs, such as transportation or being able to contact their provider. They also co-led a number of projects that served to improve patient ability to understand and negotiate both their cancer treatment and the system. In one local network, PLC and clinicians designed and provided group information sessions for patients starting treatment to help them understand the process, anticipate problems and know how to respond, a best practice that was then shared within the national PLC committee. Similar sessions for patients coming to the end of treatment were also under development.

Within local networks, work to design tumour-specific trajectories increased reliance on PLC experience, thereby enhancing their influence on managing knowledge and relations. Trajectory design involves understanding, from the patient’s perspective, all steps from investigation of a suspicious lesion to life beyond active treatment in order to coordinate among providers, set priorities and develop solutions. Indeed, only PLC have the entire vision of the trajectory. In this exercise, PLC were seen to have essential knowledge that clinicians, managers and administrators needed to work out collaborative relationships and eliminate barriers between services. Local networks employed a variety of strategies to work with PLC in trajectory design. In one, PLC members of local committees worked with staff to design surveys and conduct small group workshops of people with the specific cancer in question to clarify the current situation, and gain insight into barriers and how these might be eliminated. A large number of PLC were involved in the process, which local managers felt would “*ensure we’re moving in the right direction given our local reality*” (clinical manager, local level).

## Discussion

This study reveals three mechanisms that interact to enable PLC participation in cancer network governance. However these mechanisms are not evenly activated in all parts of the network, nor for all governance functions.

First, the spread of PCC values, emphasized persistently by the Cancer Directorate, appears to give actors “a shared value system that will help them cooperate in a collective project” ([19], p. 26). PLC participation is a deliberate effort to operationalize ‘governance through the patient’s eyes’ within network structures. It appears as a manifestation of PCC values, while also shaping the way ‘person-centred’ is conceived in context [37]. Common values underpin relational aspects of governance in the network [38]. PLC participation in committees at local level is seen to enhance mutual recognition of each actor’s contribution, and motivates development of collaborative governance capacities within organisations [39].

Second, the structures mandated by the Cancer Directorate open up opportunities for participation that are then shaped to enhance relationships among actors and among local networks. Strategies are enacted to gradually reduce the power differentials between PLC and clinician/manager members of local committees; while some involve participation processes (i.e. assuring PLC a place on the agenda), the most impactful are opportunities for PLC to inject a perspective that challenges preconceived notions of providers. This supports findings from research on priority setting in health care that sees a process of mutual influence emerge between clinicians and patients when they are able to discuss their perspectives [40]. Working with PLC on committees gives clinicians and managers a unique opportunity to obtain feedback on the services they provide and acknowledge blind spots. There are variations in the dynamic between providers and PLC in different local committees, and changes over time in this dynamic. Some authors have found that preparation on both sides is needed to smooth the way for working together [41]. Interaction between national and local levels through the committee structures enables somewhat better alignment between “policies and capabilities found at the strategic and operational levels of health systems” ([39], p. 50). While local committee members characterise their relationship with the national level as very top down, participation offers an opportunity to connect with counterparts in other organisations and form ‘parallel networks’ where they can collaborate on specific issues. Communities of practice supported by the Cancer Directorate further increase network interaction among providers and PLC as well as between PLC. These multiple relational spaces – areas of interaction and inclusion to develop collective action [42] – in collaborative governance contribute to knowledge exchange and innovation among particular actor-groups, and to breaking down “invisible walls” between actors from different groups within the network [43]. Rodriguez and Denis [44] describe inter-organisational collaboration as paradoxical because it combines autonomy and interdependence. The combination of community of practice and committee structures may be seen as a way to accommodate this paradox: work together in a spirit of “collective learning” [45] to identify problems and interdependencies, then coordinate within actor-sets to see how each can best contribute to a collective solution.

Third, PLC knowledge about living with cancer and navigating health services is recognised in decision-making as it complements knowledge of other actors to promote collective action [46]. This mechanism is most evident in areas such as trajectory design, post-treatment supports, interaction with professionals and patient information and education that are highly dependent on PLC perspectives and where provider actors recognise their own knowledge gaps.

### PLC participation and governance functions

Governance functions refer to various practices linking mobilisation of the capacities of network actors to the achievement of collective goals [19]. The mechanisms at work in the Quebec cancer network facilitate PLC participation in some but not all governance functions. Opportunities to embody and clarify values of person-centred care facilitate participation in shaping network vision and mission; the various committees support PLC participation in managing knowledge and relationships within and across network levels. However, while the Cancer Directorate nurtures PLC participation by mandating these structures, it also limits PLC participation to governance functions that lie within the purview of these structures. Two are notably absent: monitoring and resource allocation.

Local and national committees have little influence on resource allocation decisions, which presents a challenge to acting on the input and recommendations arising from PLC participation, especially when (as in efforts such as trajectory design) a set of resources outside the local organisation is required to meet patient needs. Monitoring functions are also closely guarded and tied to a narrower view of cancer care than is held by PLC. The performance indicators used across the network are selected by a closed group at Ministry level that remains sheltered from network interactions. The lack of PLC participation here raises a contradiction that is likely to increase tension in the network over time. Notions of value in patient-centred care may differ from those arising from government concerns such as health economics [47], and some describe value-based care and patient-centred care in opposition to one another [48]. In this sense, indicators of patient experience could, if they moved in opposite directions to cost control or efficiency indicators, pose problems for national network leaders. Brown, looking at the cancer system in Ontario (Canada), considers that current indicators used to measure quality goals such as patient experience, value and equity “do not match the scope of these goals” and “include some of the poorest ratings” ([26], p. 49]).

Findings of the present study reveal that local and national levels are mobilising PLC capacities in the accomplishment of network governance. In terms described by Provan and Kenis [49], the Quebec Cancer Directorate can be seen as a “network administrative organisation” that governs the network externally. However it also acts as a lead organisation and broker, playing a key role in coordinating and sustaining the national network ([49], p. 236) and orchestrating the activity of local committees, which in turn serve as “lead organisations” to coordinate local network actors. Within this dynamic, network governance functions are assumed by a distributed set of actors, sometimes filtering down hierarchically, but also emerging from organisational and operational levels and filtering across local networks.

### Implications for practice and research

Langley and Denis, looking at innovations requiring participation by heterogeneous actors, consider “that the distributed nature of the benefits and costs of any practice needs to be understood if one wants to implement change” ([50], p. i44). PLC participation appears in the present study as a promising means of ensuring that the benefits and disadvantages to patients of a given practice become part of this negotiation. However, this in-depth study over six years reveals that PLC participation in network governance is itself a complex problem. In all likelihood, it will become even more complex as participation matures, considering the paradox of interdependency and autonomy in decision-making, as well as the emerging tension highlighted by Brown [26] between patient experience and other measures of performance. In practice, results of the study suggest that PLC participation in governance takes shape through a balance between normative prescription from the network lead, and support for learning, collaboration and constant practice adaptation.

There is increasing interest in enhancing patient and citizen participation in co-producing healthcare services [26]. In Quebec, a university-led initiative is seeking to move beyond patient-centred care and integrate patients as partners in care, health professional education and health research [51]. In Europe, a major project (COGOV) is now exploring how public agencies can “exploit the drivers – and overcome the barriers – to the co-production and co-creation of innovative public value outcomes” by recognizing citizen input into the process of public governance ([13], p. 44). The present study suggests that this ambition goes hand in hand with network integration and governance structures that bring actors, including PLC, together so they can figure out how each might tailor their contribution to best meet collective goals.

Data collection was completed a few months prior to the global Covid-19 pandemic, which had an immediate and important adverse effect on the context for activating the mechanisms identified, specifically opportunities for participation and possibilities for knowledge exchange. It is hoped that the findings of this study will help guide the adjustment phase by challenging actors to find ways of activating mechanisms that support PLC participation in new forms.

### Study strengths and limitations

This case study provides an unprecedented exploration of PLC participation as it evolves in network governance structures at multiple levels. It contributes to filling an important knowledge gap around the sustainability of patient participation [52], as well as furthering understanding of factors that impact the participation of PLC in integrated cancer network governance dynamics. This aligns with the collaborative governance framework [21] and reveals it as a promising route to further explore the participation of PLC in cancer networks.

A major strength of this study is the credibility brought by detailed data collected over six years that enables exploration of a complex phenomenon: PLC participation in network governance. Informed by a conceptual framework based on governance functions [19] and collaborative governance [21], adapted to cancer care, the present study offers evidence on how PLC participation takes shape though the activation of certain mechanisms. In Quebec, PLC participation is seen to contribute to shifting top-down decision-making cultures to collaborative network governance that includes PLC [53]. One limitation of the study may be that, in collecting and analyzing data from focus groups with PLC members of local committees, we did not explore the interactions between participants during the focus groups, which may have revealed additional information.

A number of strategies were used to assure the credibility and reliability of findings [34, 54]. Analysis drew on a combination of knowledge from the literature and prolonged research team engagement, which helped prevent premature conclusions. The diversity of knowledgeable informants and data collection methods meant that multiple perspectives were integrated into the analysis. Iterative co-analysis of data and regular discussion among researchers mitigated risks of researcher subjectivity.

Activation of the mechanisms identified is highly context dependent, which limits the transferability of findings. Transferability is an inherent limitation of the case study approach [28, 55]. But by focussing on underlying mechanisms revealed in patterns emerging from natural settings, and on linkages between these and governance functions in action, researchers may assess the transferability of the study findings to other initiatives aimed at achieving PLC participation in national cancer programs. An important goal for future research would be to discover why a specific mechanism [27] is activated (or not) in order to build a theory of the intervention (PLC participation in network governance). Studying context-mechanism-outcome configurations [56] appears a logical direction for this type of explanatory research.

## Conclusions

There is growing political will to engage patients in governance structures at all levels of health systems to increase responsiveness to evolving needs [10, 57]. The Quebec Cancer Directorate’s adoption of patient-centred care as an objective, and its mandate that PLC be included in network governance structures, provides a unique opportunity to advance knowledge on the challenges and opportunities for translating political will into better care. The long study period and wide range of informants increase confidence in findings that (1) consistently emphasizing patient-centred care as a network objective, (2) assuring the flexibility, time and support to shape the mandate for PLC representation into meaningful participation, and (3) recognizing the distinct knowledge of PLC in decision-making are important means of increasing the legitimacy of PLC participation in the governance of network-based cancer care. The recursive mechanisms identified here are important to the structuration of PLC participation in dynamics of collaboration within and between levels of governance. Findings offer new theoretical and practical insight into both network governance and mechanisms that enable PLC participation to tailor and institutionalise patient-centred care within the network. Results suggest that the formalisation of committees at national and local level, and, specifically the national PLC committee, acts as a starting point in complex system change, but needs to be reinforced by mechanisms that are continually activated to sustain change over time.

## Supplementary Information

**Additional file 1.** Focus group discussion guide and interview guide.

## Data Availability

Details on the data collection and analysis grids are available upon request from the lead author (DT). Ethics considerations prevent sharing of the raw data.

## Abbreviations

PLC: People living with and beyond cancer
MSSS: Ministère de la Santé et des Services sociaux; in English: Ministry of Health and Social Services
PCC: Patient-centred care

## References

[1] Evans JM, Matheson G, Buchman S, MacKinnon M, Meertens E, Ross J, Johal H (2015). Integrating cancer care beyond the hospital and across the cancer pathway: a patient-centred approach. Healthc Q.

[2] Fashoyin-Aje LA, Martinez KA, Dy SM (2012). New patient-centered care standards from the commission on cancer: opportunities and challenges. J Support Oncol.

[3] Haward RA (2006). The Calman–Hine report: a personal retrospective on the UK’s first comprehensive policy on cancer services. Lancet Oncol.

[4] McConigley R, Platt V, Holloway K, Smith J (2011). Developing a sustainable model of rural cancer care: the Western Australian Cancer Network project. AJRH.

[5] Halabi IOS, Voz B, Gillain B, Durieux N, Odero N, Baumann A, Ziegler M, Gagnayre O, Guillaume R, Bragard M, Scholtes I, Voz B, Gillain B, Durieux N, Odero N, Baumann A, Ziegler M, Gagnayre O, Guillaume R. M et al: “Patient participation” and related concepts: a scoping review on their dimensional composition. Patient Educ Couns. 2020;103(1):5–14. 10.1016/j.pec.2019.08.001 31447194

[6] Barbazza E, Tello JE (2014). A review of health governance: definitions, dimensions and tools to govern. Health Policy.

[7] Evidence boost. a review of research highlighting how patient engagement contributes to improved care [http://citeseerx.ist.psu.edu/viewdoc/download?doi=10.1.1.676.3590&rep=rep1&type=pdf]

[8] Ziebland S, Coulter A, Calabrese JD, Locock LE (2013). Understanding and using health experiences: improving patient care.

[9] Bombard Y, Baker GR, Orlando E, Fancott C, Bhatia P, Casalino S, Onate K, Denis J-L, Pomey M-P. Engaging patients to improve quality of care: a systematic review. Implement Sci 2018;13(1):1-22. 10.1186/s13012-018-0784-z PMC6060529 30045735

[10] Epstein RM, Fiscella K, Lesser CS, Stange KC (2010). Why the nation needs a policy push on patient-centered health care. Health Aff (Millwood).

[11] Bodolica V, Spraggon M, Tofan G (2016). A structuration framework for bridging the macro–micro divide in health-care governance. Health Expect.

[12] Castro EM, Van Regenmortel T, Vanhaecht K, Sermeus W, Van Hecke A (2016). Patient empowerment, patient participation and patient-centeredness in hospital care: a concept analysis based on a literature review. Patient Educ Couns.

[13] Co-production and. co-governance: strategic management, public value and co-creation in the renewal of public agencies across Europe. Deliverable 1.1: literature review [http://cogov.eu/wp-content/uploads/2020/05/COGOV-DeliverableWP1-1.pdf].

[14] Programme québécois de lutte contre le cancer. Pour lutter efficacement contre le cancer, formons équipe http://publications.msss.gouv.qc.ca/acrobat/f/documentation/1997/97-729-5.pdf.

[15] Les personnes touchées par. le cancer: partenaires du réseau de cancérologie - Cadre de référence http://publications.msss.gouv.qc.ca/msss/document-000346/.

[16] Plan directeur en. cancérologie 2013–2015 - Ensemble, en réseau, pour vaincre le cancer http://publications.msss.gouv.qc.ca/msss/document-000346/.

[17] Ferlie E, Fitzgerald L, McGivern G, Dopson S, Bennett C (2013). Making wicked problems governable? The case of managed networks in health care.

[18] Hatchuel A, Heurgon E, Landrieu J (2000). Prospective et gouvernance: quelle théorie de l’action collective?. Prospective pour une gouvernance démocratique.

[19] Denis JL, Champagne F, Pomey MP, Préval J, Tré G (2005). Toward a framework for analysis of governance in healthcare.

[20] Tremblay D, Touati N, Roberge D, Breton M, Roch G, Denis JL, Candas B, Francoeur D (2016). Understanding cancer networks better to implement them more effectively: a mixed methods multi-case study. Implement Sci.

[21] Emerson K, Nabatchi T, Balogh S (2011). An integrative framework for collaborative governance. J Public Adm Res Theory.

[22] Tremblay D, Touati N, Poder T, Vasiliadis H-M, Bilodeau K, Berbiche D, Denis J-L, Pomey M-P, Hébert J, Roch G (2019). Collaborative governance in the Quebec Cancer Network: a realist evaluation of emerging mechanisms of institutionalization, multi-level governance, and value creation using a longitudinal multiple case study design. BMC Health Serv Res.

[23] Addicott R, McGivern G, Ferlie E (2006). Networks, organizational learning and knowledge management: NHS cancer networks. Public Money Manag.

[24] Souliotis K, Peppou L-E, Tzavara C, Agapidaki E, Varvaras D, Buonomo O, Debiais D, Hasurdjiev S, Sarkozy F (2018). Cancer patients’ organisation participation in heath policy decision-making: a snapshot/cluster analysis of the EU-28 countries. BMJ Open.

[25] Ferlie E, Mcgivern G, FitzGerald L (2012). A new mode of organizing in health care? Governmentality and managed networks in cancer services in England. Soc Sci Med.

[26] Brown A (2015). The challenge of quality improvement at the system level. Whither CCO?. Healthc Q.

[27] Astbury B, Leeuw FL. Unpacking black boxes: mechanisms and theory building in evaluation. Am J Eval 2010;31:363-81.

[28] Stake RE (2013). Multiple case study analysis.

[29] Mills AJ, Durepos G, Wiebe EE (2009). Case within a case. Encyclopedia of case study research.

[30] Guest G, Namey EE, Mitchell ML (2013). collecting qualitative data: a field manual for applied research.

[31] Nag R, Gioia DA (2012). From common to uncommon knowledge: foundations of firm-specific use of knowledge as a resource. AMJ.

[32] Green J, Thorogood N (2009). Group interviews and discussions. Qualitative Methods for Health Research.

[33] LePan C (2013). Review of QDA Miner. Soc Sci Comput Rev.

[34] Miles MB, Huberman M, Saldana J (2020). Qualitative data analysis. A methods sourcesbook.

[35] Belotto MJ (2018). Data analysis methods for qualitative research: managing the challenges of coding, interrater reliability, and thematic analysis. Qual Rep.

[36] Gioia DA, Kevin G, Corley A, Hamilton L (2013). Seeking qualitative rigor in inductive research: notes on the Gioia methodology. Organ Res Methods.

[37] Waters RA, Buchanan A (2017). An exploration of person-centred concepts in human services: a thematic analysis of the literature. Health Policy.

[38] Willem A, Gemmel P (2013). Do governance choices matter in health care networks? An exploratory configuration study of health care networks. BMC Health Serv Res.

[39] Denis JL, Usher S. Governance must dive into organizations to make a real difference: comment on” Governance, government, and the search for new provider models”. IJHPM. 2016;6(1):49–51. 10.15171/ijhpm.2016.89 PMC5193506 28005542

[40] Boivin A, Lehoux P, Lacombe R, Burgers J, Grol R (2014). Involving patients in setting priorities for healthcare improvement: a cluster randomized trial. Implement Sci.

[41] Pomey M-P, Lebel P, Clavel N, Morin E, Neault C, Tétreault B, Mulliez A (2018). Development of patient-inclusive teams: toward a structured methodology. Healthc Q.

[42] Kellogg KC (2009). Operating room: relational spaces and microinstitutional change in surgery. Am J Sociol.

[43] Liberati EG, Gorli M, Scaratti G (2016). Invisible walls within multidisciplinary teams: disciplinary boundaries and their effects on integrated care. Soc Sci Med.

[44] Rodríguez C, Langley A, Béland F, Denis JL (2007). Governance, power, and mandated collaboration in an interorganizational network. Adm Soc.

[45] Wenger E (1998). Communities of practice: learning as a social system. Syst Thinker.

[46] Pomey MP, Hihat H, Khalifa M, Lebel P, Néron A, Dumez V (2015). Patient partnership in quality improvement of healthcare services: patients’ inputs and challenges faced. Patient Exp J.

[47] Porter ME, Teisberg EO (2006). Redefining health care: creating value-based competition on results.

[48] Tseng EK, Hicks LK (2016). Value-based care and patient-centered care: divergent or complementary?. Curr Hematol Malig Rep.

[49] Provan KG, Kenis P (2007). Modes of network governance: structure, management, and effectiveness. J Public Adm Res Theory.

[50] Langley A, Denis JL (2011). Beyond evidence: the micropolitics of improvement. BMJ Qual Saf.

[51] Dumez V, Pomey MP. From medical paternalism to care partnerships: a logical evolution over several decades. In: Pomey MP, Denis JL, Dumez V, editors, Patient engagement: how patient-provider partnerships transform healthcare organizations. Cham: Palgrave Macmillan; 2019. p. 9–16.

[52] Berger Z (2018). Metrics of patient, public, consumer, and community engagement in healthcare systems: How should we define engagement, what are we measuring, and does it matter for patient care? Comment on ‘Metrics and evaluation tools for patient engagement in healthcare organization- and system-level decision-making: a systematic review. Int J Health Policy Manag.

[53] Tremblay D, Latreille J, Bilodeau K, Samson A, Roy L, L’Italien M-F, Mimeault C (2016). Improving the transition from oncology to primary care teams: a case for shared leadership. J Oncol Pract.

[54] Wu YP, Thompson D, Aroian KJ, McQuaid EL, Deatrick JA (2016). Writing and evaluating qualitative research reports. J Pediatr Psychol.

[55] Yin RK (2011). Qualitative research from start to finish.

[56] Realist, Evaluation. https://www.communitymatters.com.au/RE_chapter.pdf.

[57] Gagliardi AR, Legare F, Brouwers MC, Webster F, Wiljer D, Badley E, Straus S (2011). Protocol: developing a conceptual framework of patient mediated knowledge translation, systematic review using a realist approach. Implement Sci.

[58] Tri-Council policy. statement: ethical conduct for research involving humans [https://ethics.gc.ca/eng/documents/tcps2-2018-en-interactive-final.pdf]

