# Supplementary material for: Patient participation in cancer network governance: a six-year case study
Source: BMC Health Serv Res. 2021 Sep 7;21:929. doi: 10.1186/s12913-021-06834-1 (PMC8423332; doi:10.1186/s12913-021-06834-1)
Supplement: Supplementary file 1 — Additional file 1. Focus group discussion guide and interview guide. [file 12913_2021_6834_MOESM1_ESM.docx]

**Additional file 1: Focus group discussion guide and interview guide**

PLC focus group procedure and discussion guide

The researchers conducted focus groups with people living with and beyond cancer (PLC) to better understand their experience of participating on local governance committees in the cancer network. Collaborators at each study site played a role in participant recruitment by informing local PLC representatives of the study and describing reasons they might wish to participate. The collaborators provided the research team contact information for interested parties, and the research coordinator (LL) issued invitations. The focus groups had a double justification. The first, related to data collection for the study, was to benefit from a range of perspectives on the current situation by stimulating discussion and exchange. The second, especially important given the different length of experience of many local PLC representatives, was to create a learning opportunity among PLC committee representatives around this relatively new role. The following sections describe: 1) the practical dimensions of conducting the focus group with PLC, adapted from Guest et al (2013)^1^, followed by the discussion guide; and 2) the interview guide for individual interviews based on Emerson's collaborative governance framework^2^.

**Section 1: Focus group practical dimensions and interview guide**

**1. On site briefing of the research team**

The facilitator, co-facilitator, observer and, where available, local contact met before the start of the focus group to review the procedure and their respective roles

**2. Focus group location**

The focus groups were held in meeting rooms reserved in the cancer clinics.

**3. Process: Setting the tone and expectations**

The most senior member of the research team (DT) welcomed participants upon arrival, thanked them for participating and answered any preliminary questions. When all had arrived, she invited them to take their seats.

Participants had no questions at this time and were clear on the reason for their participation in the focus group.

The co-facilitator (LL) - research coordinator within the research team - who had contacted participants, introduced the focus group procedure and objectives, as well as the role she and the observer (a Masters' student) would play during the discussion as note-takers to ensure that all the information participants provided was included in the study.

She then introduced the facilitator (DT) who would lead the discussion.

The co-facilitator reviewed the objectives of the study and explained how the focus group would proceed, reviewing key elements of the written consent form approved by the ethics board:

- The study objective is to better understand the participation of people living with and beyond cancer (PLC) in governance committees within establishments that are part of the cancer network (local level) and at national level (National PLC Committee).
- We want to hear from you about what works well and less well in the opportunities available at both levels for you to participate in guiding system improvements.
- The discussion will last about an hour and a half and there will be a break half way through for refreshments.
- Your participation will not bring you any immediate benefit, other than contributing your knowledge of the organization of cancer care services to influence their improvement.
- The discussion may provoke an emotional response as you discuss your experience and we are respectful of this.
- Confidentiality is paramount and we assure you that no personal information you provide today will be shared outside this room. No names will be used if we include a statement made during discussion in the final article.
- The discussion will be audio recorded, but when we transcribe it, we will remove all names and replace them by codes. It will not be possible for anyone to associate a statement with an individual person.
- In that spirit, we ask for your cooperation in ensuring that "what's said in this room, stays in this room". Please refrain from talking about today's discussion and revealing the identities of other participants.

4. Participant comfort with discussing their experience and review of study ethics

The co-facilitator asked if there were any questions and asked participants to read and sign the information and consent forms (these were pre-signed by the lead researcher).

Participants were clear about elements of the consent form. It was important to state that the research team had no contact with PLC care teams.

The co-facilitator then detailed a number of rules to be respected during the discussion:

- Confidentiality of discussion content and participant identities
- Non-judgment: there are no right or wrong answers
- Relevance and consideration of time: participants are asked to keep the discussion general and not enter into personal details of care received.
- Interactivity: participants are encouraged to put questions to other participants or offer complementary or contrasting input

The co-facilitator described the facilitator's role as stimulating discussion relevant to the study objectives and ensuring that all participants have a chance to express their thoughts. She then introduced the facilitator and co-facilitator.

The facilitator recalled the study objectives and presented the cancer trajectory (supported by a poster displayed on the wall) and the elements to be discussed around the governance and integration of services.

The facilitator introduced the idea of governance as the set of activities and decisions that ensures people have access to care, that there is continuity of care (that people do not fall through the cracks), and that care meets the whole of range of needs people experience along the cancer trajectory. She emphasized: Patients like you have a deep knowledge of the cancer network gained from your experience over the trajectory and this knowledge has a valuable role in identifying the network's strengths and weaknesses.

**5. Discussion guide**

The facilitator used seven questions, drawing on the study's conceptual framework (based on collaborative governance), to guide the focus group discussion.

1. What is your understanding of the cancer care network in your region/establishment?
2. What is your understanding of the role of PLC in (governance) committees in the cancer network (regional and national)?
3. What is your perception of the credibility and influence of PLC within these committees?
   1. To what extent can PLC influence discussion and decisions?
   2. Can you provide examples where PLC influenced decisions?
4. To what extent are you informed of decisions that are made?
5. To what extent are you able to participate in implementing these decisions?
6. To what extent do you think that the difficulties patients encounter are considered by the committees that govern the network?
7. Overall, what are your thoughts about the way the network is coordinated, the way different people (clinicians, managers, government) communicate with each other and obtain information they need?

**Section 2: Interview guide, individual interviews**

Individual interviews were conducted with policymakers (17), managers (15), clinician managers (19), clinicians (19) and PLC (3, on national level committees). All are knowledgeable participants involved in network governance committees at the local or national level. They are key informants regarding the participation of PLC, which is promoted by the national cancer program.

The interview guide of open-ended questions was developed based on the study's conceptual framework, with probes specifically related to Emerson's collaborative governance framework.

Individual interview guide

| **Open-ended question** | **Probes** | **Dimension of Emerson's collaborative governance framework** |
| --- | --- | --- |
| Context: local issues around improving the quality of life and experience of care of PLC, and responses to these issues | 1. What collective actions have been undertaken? 2. Which are most promising? 3. What motivated these actions? | System context |
| Engagement within governance structures (local / national) | 1. To what extent are actors and stakeholders involved in governance structures? 2. Within these governance structures, are there mechanisms to discover the concerns of different parties? 3. How does a common vision of shared objectives take shape? 4. How are controversies revealed? 5. What procedures are in place to assure proper functioning of the network? 6. How does the network ensure actor engagement in decisions made by the governance committee? | Relate to collaborative governance dimensions of engagement:  b) Discovery,  c) Definition,  d) Deliberation,  e) Procedural decisions,  f) Substantive determination |
| Dynamics within governance structures | 1. Can you describe how trust has or has not been developed within governance structures 2. To what extent do actors understand each other's positions (even if they don't agree)? 3. Can you describe how actors develop credibility within governance structures? 4. What is your perception of the credibility of PLC representatives on the committee? Do they influence discussion and decisions? Can you provide examples? 5. Do you feel the cancer network in your region is considered credible by stakeholders? 6. To what extent are actors committed to collaborative action that extends beyond silos | Relate to collaborative governance dimensions of shared motivation:  b) Mutual understanding  c, d, e) Legitimacy  f) Commitment |
| Development of capacity for joint action | 1. What agreements are in place to operationalize collaboration? 2. What collaborative actions have led to the emergence of new leadership roles? 3. How does the network promote knowledge exchange? | Relate to collaborative governance dimensions of joint action:  a) Institutional arrangements  b) Leadership  c) Knowledge |
| Impact of collaborative governance activities | 1. To what extent do monitoring and control mechanisms enable the documentation of collaborative initiatives and their impact on PLC? | Relate to impacts on collaborative dynamics and added value |
| Adaptation of networks and organizations | 1. What unanticipated effects have you observed? 2. How have the mission, management practices, governance practices, resource distribution and evaluation changed? | Relate to collaborative governance dimension of adaptation |

^1^ Guest G, Namey EE, Mitchell M. Focus groups. *Collecting Qualitative Data: A Field Manual for Applied Research*. 2013;55:172-222.

^2^ Emerson K, Nabatchi T. *Collaborative Governance Regimes*. Georgetown University Press; 2015.
